# The Stability, Rheological Properties and Interfacial Properties of Oil-in-Water (O/W) Emulsions Prepared from Dielectric Barrier Discharge (DBD) Cold Plasma-Treated Chickpea Protein Isolate and Myofibrillar Protein Complexes

**DOI:** 10.3390/foods12193629

**Published:** 2023-09-29

**Authors:** Dianbo Zhao, Yanfang Zhou, Lixue Sun, Jinfeng Tian, Qisen Xiang, Ke Li

**Affiliations:** Henan Key Laboratory of Cold Chain Food Quality and Safety Control, College of Food and Bioengineering, Zhengzhou University of Light Industry, Zhengzhou 450001, China; 2006032@zzuli.edu.cn (D.Z.); ungzyf@163.com (Y.Z.); 14747591208@163.com (L.S.); tianjinfeng@saishangmilk.com (J.T.); xianqisen2006@163.com (Q.X.)

**Keywords:** myofibrillar protein, chickpea protein isolate, cold plasma, emulsion stability, interfacial properties

## Abstract

In order to increase the development and utilization of chickpea protein isolate (CPI) and improve the stability of myofibrillar protein (MP) emulsions, the effect of dielectric barrier discharge (DBD) plasma-modified CPI on the emulsifying properties of MP was investigated. Three different O/W emulsions were prepared using MP, MP + CPI complex, or MP + DBD-treated CPI complex as the emulsifier. Compared with the emulsion prepared from MP, the emulsifying activity index and stability of DBD-treated CPI and MP complex (MP + CPI_DBD_) were increased (*p* < 0.05) from 55.17 m^2^/g to 74.99 m^2^/g and 66.31% to 99.87%, respectively. MP + CPI_DBD_ produced more stable emulsions with the lowest Turbiscan stability index (TSI) values for a given 3600 s. At shear rates from 0 to 1000^−1^, MP + CPI_DBD_-stabilized emulsions had higher viscosities, which helped to reduce the chance of aggregation between oil droplets. The optical microscope and particle size distribution of emulsions showed that MP + CPI_DBD_ emulsions had the lowest droplet size (d_4,3_) and exhibited more uniform distribution. MP + CPI_DBD_ emulsions had lower interfacial tension. DBD pretreatment increased the adsorbed protein content in the emulsion stabilized by MP + CPI_DBD_ as compared to the MP + CPI complex and promoted the adsorption of CPI by higher ratios of adsorbed proteins as indicated by its intensity in SDS-PAGE. Scanning electron microscopy confirmed that the emulsion prepared from MP + CPI_DBD_ had smaller particle size and more uniform dispersion. Therefore, using DBD-modified CPI could enhance the stability of MP emulsions.

## 1. Introduction

The quality of meat products is greatly influenced by myofibrillar protein (MP), which serves as the primary protein in the meat processing industry. The fat globules in an emulsified meat system have the ability to adsorb MP on their surface [1]. On the other hand, MP on the surface of the fat sphere interacts with the matrix protein to form an elastic gel during the heating process to ensure the textural properties and water retention of the product [2]. The content of MP in the whole muscle protein system accounts for 50–60% and it can form >90% of the emulsifying capacity of the whole meat product [3]. Studies have shown that when the fat content is about 20–30% in emulsion-type meat products, MP is adsorbed on the surface of oil droplets to wrap fat particles, making the protein emulsion system relatively stable [4]. Therefore, in most cases, the emulsion-type meat products can be regarded as an emulsion gel system composed of a protein emulsion [5].

However, it is difficult to ensure the long-term stability of an emulsifying system by relying on protein alone. In addition, there are certain factors affecting the stability of a protein emulsion during storage, such as flocculation, coalescence, austenization, and phase transformation [6]. These phenomena lead to the instability of the protein emulsion as water and oil leakage from the product easily occur, which affects the appearance and reduces the edible quality of the product, and even endangers the shelf life of the food [7]. Therefore, enhancing the functional properties of MPs (especially their gel properties) is the key to improving the quality of meat. On the one hand, we can improve the properties of a protein emulsion by modifying it via various techniques. On the other hand, protein compounds with other substances are a valuable approach to enhance the emulsification stability of protein. This approach mainly concentrates on adding emulsifying agents such as polysaccharides and proteins to enhance the emulsifying stability [8]. According to previous study findings, the introduction of polysaccharides to whey protein led to a rise in emulsion viscosity, a decrease in particle mobility within the emulsion, and an enhancement in the emulsion’s physical stability [9]. Jiang et al. [10] showed that adding appropriate pea protein hydrolysates into an O/W emulsion could help increase the density and thickness of the oil–water interface film, inhibit the emulsification behavior, and improve the emulsification stability of the emulsion. Ji et al. [11] conducted a study to analyze the physical and chemical properties of a nano-emulsion made by combining sodium caseinate (SC) and soy protein isolate (SPI). Their findings indicated that this nano-emulsion possessed favorable characteristics and could maintain its stability over extended periods of time. Consequently, it can be considered as an appropriate liposoluble nutrition delivery system for clinical use.

Chickpea is widely consumed around the world and has great potential for development due to its rich nutritional content [12]. Chickpea has become the focus of attention due to its high protein content (18.89–28.75%). Compared with soybean, it offers the benefits of substantial production quantities, economical pricing, exceptional equilibrium in its structure of vital amino acids, remarkable bioavailability, and minimal allergenicity [13]. Currently, with the increasing consumption of plant proteins, CPI is gaining more attention as a sustainable and healthy ingredient in food formulations [14]. However, due to the tight structure of natural protein, it rarely shows good functional characteristics, which affects the efficiency of protein utilization. It must therefore be modified to meet the needs of food processing and the development of new products.

As a new non-thermal processing technology, plasma technology shows great potential in protein functional modification. It has low energy consumption, can avoid the loss of food quality caused by traditional modification methods, and has already been applied in the food industry [15]. Bußler et al. [16] showed that the protein solubility of pea protein isolate was increased to 191% after DBD plasma treatment for 10 min. Ji et al. [17] reported that the average particle size of peanut protein decreased from 1112.7 nm to 1062.3 nm and the emulsifying properties were also enhanced by plasma treatment for 1–3 min. In the early stage, we used cold plasma to treat chickpea protein isolate (CPI) for 4 min, and we found that the high-energy particles released by plasma destroyed the intermolecular forces, the particle size decreased, and the solubility of the protein increased by 22.88% [18]. Concurrently, the protein was slightly oxidized by the plasma, which allowed protein molecules to be more firmly adsorbed on the surface of the oil droplets, increasing the emulsifying stability of the protein.

Under the dual effects of consumers’ increasing health requirements and their increasing consumption of animal protein on resources and the environment, the substitution of animal protein with plant protein in food formulas has gained increasing attention. The mixed protein system can further expand the application range of plant proteins on the basis of giving full play to the nutritional advantages of plant proteins, while bringing some unique functional characteristics to products and reducing production costs [19]. At present, the interaction between animal and plant proteins is still poorly understood. In this paper, we prepared emulsion by mixing MP and CPI that had been treated or untreated by dielectric barrier discharge. The physical and chemical properties and interface properties of the emulsions were studied in detail to provide a theoretical reference for improving the quality and nutritional value of related foods and broadening the application range of plant proteins.

## 2. Materials and Methods

### 2.1. Materials

Kabuli chickpeas were purchased from Guyuan County, Ningxia Autonomous Region, with a chemical composition including 14.1% protein, 2.7% fat, 48.1% starch, and 17.6% dietary fiber. Jinlongyu soybean oil and fresh chicken breast were purchased from Dennis supermarket (Zhengzhou, Henan, China). The chicken breasts were minced through a grinder (JR-120, Shandong, China) fitted with a plate having 6 mm diameter holes. Then, they were mixed well, packed into vacuum-packed bags (thickness of 0.08 mm, polyethylene material) at approximately 400 g per bag, and then vacuumed (FW-3150, Fresh World Electrical Appliances Co., Ltd, Guangzhou, China). The meat samples were stored in a freezer at −20 °C and used within 2 weeks. All other reagents and chemicals used were of analytical grade.

### 2.2. Preparation of Emulsions with a Mixed Protein System

#### 2.2.1. Preparation of Chickpea Protein Isolate

The preparation of CPI was performed via a previous method described by Wang et al. [20] with a few minor modifications. The chickpeas were ground into a powder (CLF-30B, HL, China). Then, the powder was sieved through an 80-mesh sieve and dried (BGZ-30, Boxun, Shanghai, China) in an oven at 50 °C until constant weight. Thereafter, they were mixed with n-hexane in a ratio of 1:10 and then stirred at room temperature for 2 h to degrease. After standing for 3 h, the supernatant was decanted, and the precipitate was then extracted with n-hexane again. Defatted chickpea powder was obtained by repeating the process three times and removing excess n-hexane in a fume hood. Afterwards, the powder was mixed with deionized water in a ratio of 1:8, and the pH of the mixture was adjusted to 9.0 using 1 M NaOH. The mixture was then centrifuged (AvantiJ-26SXPI, Beckman Coulter, West Sacramento, CA, USA) for 20 min at 6000 rpm. The obtained supernatant had a pH of 4.9, which was adjusted using 1 M HCl. Centrifugation was carried out again under the same centrifugation conditions. The obtained precipitate was mixed with deionized water, and the pH was regulated to 7.0 by using 1 M HCl. Finally, the resulting solution was dialyzed and freeze-dried (Lab-1–50, Boyikang Experimental Instrument Co. LTD., Beijing, China) to obtain CPI. Dialysis reduced the conductivity of CPI_DBD_ to less than 15, the CPI_DBD_ was pre-frozen in the freeze dryer for 3 h, then vacuumed to a chamber vacuum of about 40 Pa. After tightening the valve, CPI was obtained by continued freeze drying for 15 h.

Subsequently, CPI was dissolved in deionized water (160 mL, 3%, *w*/*w*). After stirring at room temperature for 2–3 h, the solution was stored at 4 °C overnight (in order to ensure complete hydration). Then, the CPI dispersions were treated using plasma equipment (APM-400M, PSM, Seongnam-si, Republic of Korea). The carrier gas was nitrogen, the flow rate was 350 L/min, the electrode temperature was 75 °C, and the voltage was 8 kV. Twenty-milliliter volumes of solution were taken and treated for 4 min. After balancing the temperature of the solutions, they were freeze-dried.

#### 2.2.2. Preparation of Chicken MP

MP was taken from chicken meat following a procedure detailed in a previous study [21]. The abovementioned minced chicken breasts were thawed for 12 h at 4 °C. Then, they were chopped into a meat paste using a Waring Blender (GM200, Laichi, Haan, Germany) at a speed of 2000 rpm for 30 s, with a pause of 10 s between each interval. The obtained meat was stored at a temperature of 4 °C for MP extraction on the same day. To extract MP, a cooling isolation buffer (10 mM Na_2_HPO_4_/NaH_2_PO_4_, 2 mM MgCl_2_, 0.1 mM NaCl, 1 mM EGTA, pH 7.0, 4 °C) was utilized. The mixture was homogenized for 60 s at 10,000 rpm, in 20 s intervals. After filtration using a 20-mesh filter, the mixture was centrifuged for 20 min at 2000× *g* to obtain the precipitate. The above steps were repeated three times. Then, 4 times the volume dose of 0.1 M NaCl was added to purify the protein, the mixture was centrifuged under the same centrifugal conditions, and the supernatant was discarded. This operation was repeated three times. Before the final centrifugation, the mixture was filtered through three layers of gauze, centrifuged to obtain purified myofibrillary protein, and stored at 4 °C for later use. The whole protein extraction process was carried out at 0–4 °C. Bovine serum protein was used as the standard protein to draw the standard curve, and the concentration of myofibrillary protein was determined via the Biuret method [21].

#### 2.2.3. Emulsion Preparation

First, 1.5% (*w*/*w*) CPI and CPI_DBD_ samples were stirred for 2 h and the concentration of MP was harmonized (Ultraturrax T25, IKA, Staufen, Germany) to 6 mg/mL using phosphate buffer (20 mmol/L Na_2_HPO_4_/NaH_2_PO_4_, 0.6 mol/L NaCl, pH 7.0). Second, mixtures of protein and soybean oil were added into a glass cylinder with a diameter of 40 mm at a ratio of 3:1 (*w*/*w*). Lastly, the emulsion of the mixed system was homogenized at 10,000 rpm 4 times for 30 s each time. The obtained emulsion was stored at 4 °C for later use. For subsequent convenience, the emulsions were named MP, MP + CPI, and MP + CPI_DBD_.

### 2.3. Emulsifying Activity Index (EAI) and Emulsifying Stability Index (ESI)

The emulsions were then subjected to EAI and ESI determination as described by Li et al. [22] with a few modifications. Briefly, 50 μL of freshly prepared emulsion was obtained from the bottom immediately after homogenization and dispersed in 5 mL of 0.1% sodium dodecyl sulfate (SDS). An ultraviolet spectrophotometer (TU-1810, Persee, Beijing, China) was used to read the absorbance at 500 nm, which was recorded as A_0_. The EAI is defined as follows:

EAI (m^2^/g) = (2.303 × 2 × A_0_ × N)/(C × Φ × 10,000).

The ESI is defined as follows:

ESI (%) = A_10_/A_0_ × 100%.

A_10_ represents the absorbance at 10 min. The dilution factor is represented by N, the protein concentration before emulsification is represented by C (g/mL), and the oil volume fraction of the emulsion is represented as Φ.

### 2.4. Turbiscan Stability Index (TSI)

The stability of the newly formed emulsions was analyzed using a Turbiscan Lab Expert analyzer (Formulaction, Toulouse, France) following the methodology described by Li et al. [23]. Mixed protein emulsions were prepared according to the method described in Section 2.2.3, and 18 mL of emulsion was taken for test scanning. The test procedure involved scanning once every 60 s, and the total scanning time was 3600 s. In this study, since the sample was opaque to light, the change in the backscattered light intensity signal of the sample could be used to calculate the TSI of the emulsion system.

### 2.5. Optical Microscopy

The microstructure of the fresh emulsions was observed via optical microscopy. The emulsions were diluted to 1 mg/mL with a phosphate buffer, and 10 μL of sample was placed in the center of the slide. The cover glass was placed slowly to prevent bubbles from forming. The microstructure of the emulsions was observed and images were obtained under a 50× objective lens using an optical microscope (PH 50, Phenix, Beijing, China).

### 2.6. Particle Distribution and Size Measurement

The emulsions were analyzed for their particle distribution and size according to the procedure detailed by Jahromi et al. [24] with slight modifications. A Mastersizer (LS 13320, Beckman Coulter, West Sacramento, USA) was used to determine the particle size and distribution of the emulsions, and the results are represented by the mean volume diameter d_4,3_. All measurements were performed three times.

### 2.7. Creaming Index (CI)

The fresh emulsions were transferred into glass test tubes and stored at 4 °C. The emulsions were photographed at 0, 12, 24, 36, 48, and 60 h for apparent stability analysis. In addition, the initial height of the emulsion (H_0_) and the height of the aqueous phase at different moments (H_t_) were recorded, and the emulsion index was calculated using the formula CI (%) = H_0_/H_t_ × 100%.

### 2.8. Rheological Property Analysis

To determine the apparent shear viscosity, a dynamic rheometer (Discovery HR-1, TA, Chicago, IL, USA), equipped with parallel plates 40 mm in diameter, was used to conduct frequency scan tests and temperature scan measurements of the emulsions [21,25]. Three parallel repeat measurements were performed.

#### 2.8.1. Apparent Viscosity

Fresh emulsions were transferred via the method in Section 2.5; emulsion samples were placed between parallel plates at 25 °C, and the gap was set to 0.4 mm. A range of shear rates from 0 to 1000 s^−1^ was used to measure the apparent shear viscosity of the emulsions.

#### 2.8.2. Frequency Sweep Testing

Frequency sweep measurements were used to characterize the changes in the storage modulus (G’) and loss modulus (G″) of the sample emulsions with angular frequency. The test frequency ranged from 0.1 to 100 rad/s, and the shear strain was 0.5%.

#### 2.8.3. Temperature Sweep Measurements

Temperature sweep measurements of the emulsion were performed at a shear strain of 0.5%. After the emulsion was placed on the test plate, the edge was sealed with silicone oil to prevent moisture evaporation during the test. The temperature ramp program was run at scan rates of 2 °C/min and 5 °C/min, respectively, from 20 °C to 90 °C, and then from 90 °C to 20 °C again. During the dynamic rheological measurements, G′ and G″ were recorded.

### 2.9. Interfacial Properties

#### 2.9.1. Interfacial Tension

The interfacial tension was measured according to O’Sullivan et al. [26] with some modifications. The interfacial tension between the protein solution and oil phase was measured via the platinum plate method and characterized using an interfacial tensiometer (K100, Krüss, Hamburg, Germany). The experiment was conducted for 3600 s, and the temperature was maintained at 20 °C for the duration of the experiment.

#### 2.9.2. Surface Protein Adsorption

The contents of adsorbed proteins and non-adsorbed proteins in the emulsions were determined according to a method used in former studies, with minor modifications [23,27]. Briefly, freshly prepared emulsions (10 mL) were centrifuged at 12,000 rpm for 30 min at 4 °C. The aqueous solution was collected in a needle tube for later use. In order to remove non-adsorbed proteins, the emulsified layer was washed twice via dispersion in 0.6 mol/L NaCl buffer, then centrifuged at 12,000 rpm for 30 min at 4 °C. The emulsion layer was dispersed in 1% SDS solution, thoroughly mixed and frozen at −20 °C for 24 h, then thawed overnight at 4 °C and centrifuged under the same conditions. The aqueous solution (containing adsorbed proteins) was collected and the concentration of adsorbed proteins was determined using a BCA protein assay kit. The concentration of non-adsorbed protein was decided by using the distinction between the concentrations of the whole emulsion and adsorbed protein.

#### 2.9.3. Composition of Interfacial Proteins

Sodium dodecyl sulfate-polyacrylamide gel electrophoresis (SDS-PAGE) was used to determine the protein composition of adsorbed and non-adsorbed proteins in the emulsions. SDS-PAGE analysis was performed using 10% acrylamide separating gels and 5% acrylamide stacking gels. The sample loading volume was 10 μL, and electrophoresis was conducted at 80 V for 20 min and 110 V for 1.2 h. The sample was stained with Coomassie Bright Blue R-250 for 30 min, then placed in decolorization solution until the background was transparent. Images were obtained using a gel imager (XRS+, Bio-Rad, Hercules, CA, USA).

### 2.10. Microstructure

Emulsions were prepared by using the method in Section 2.5. The micro-morphology of the emulsions was observed via scanning electron microscopy (SEM) (SU 8100, Hitachi co., LTD., Tokyo, Japan). The specific operating conditions were as follows: sublimation at a temperature of −80 °C for 10 min, and then gold was sprayed on the samples, and the micro-morphology of the emulsions was observed under the condition of 5 kV.

### 2.11. Statistical Analysis

All experiments were conducted in triplicate. The data are expressed as means ± standard deviations. The SPSS 22.0 software (IBM Corporation, New York, NY, USA) was used to analyze the data with one-way ANOVA. The comparison of the means was performed using Duncan’s multiple-range tests to identify significant differences (*p* < 0.05) between means.

## 3. Results and Discussion

### 3.1. EAI and ESI

The EAI represents the adsorption capacity of protein at the oil–water interface, while the ESI reflects the ability of the protein to stabilize the emulsion within a certain period of time. Therefore, they are important indicators for the measurement of the emulsifying performance of an emulsion [28]. From Table 1, it can be seen that the emulsifying performance of the hybrid protein system was improved compared to that of MP alone (EAI 8.97 m^2^/g, ESI 66.7%). Moreover, the system MP-CPI_DBD_ had the best emulsifying properties: its emulsifying activity and emulsifying stability index values were 73.99 m^2^/g and 99.87%, respectively. Our previous study showed that the energetic particles and generated active substances can expand the structure of CPI and increase the surface hydrophobicity in the process of CPI plasma treatment [18]. The exposure of active sites would conceivably increase the CPI interacting with MP [29]. On the other hand, the reduced particle size of the mixed protein system can make protein more flexible to adsorption on the oil –water interface. Due to the above reasons, the emulsion prepared from the mixed system has better emulsifying activity and stability [30]. Zhang et al. [31] found that the electrostatic interactions between protein molecules were enhanced via the addition of whey proteins to soybean isolate proteins, and the EAI and ESI values of the emulsions were increased with the addition of whey proteins.

### 3.2. TSI

**Figure 1:**
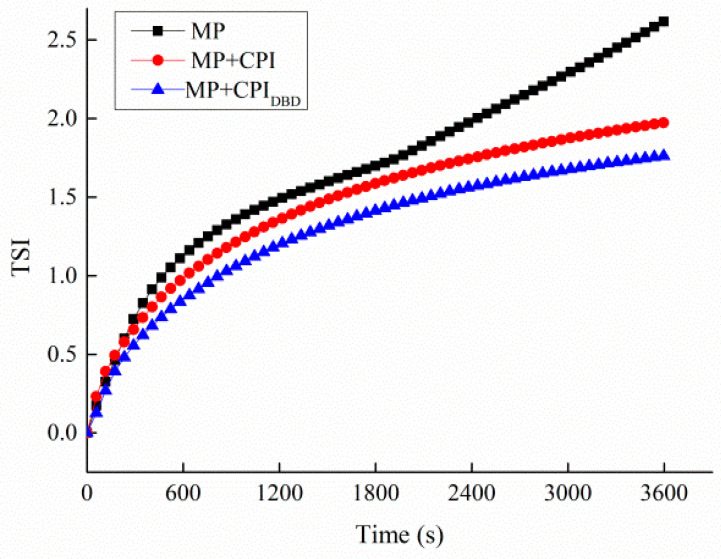
Turbiscan stability index values of emulsions prepared with myofibrillar protein (MP) and with untreated and dielectric barrier discharge (DBD)-treated CPI-MP complexes. MP: myofbrillar protein; MP + CPI: addition of native CPI; MP + CPI_DBD_: addition of DBD-treated CPI.

The TSI is a dynamic stability parameter calculated using a multi-heavy light scattering analyzer by monitoring the scattering and transmission intensity of the sample with regard to the test beam. It can comprehensively reflect the various instability processes of the emulsion during the test process, including flocculation, coalescence, and stratification. A higher TSI indicates a less stable emulsion [25,32]. Figure 1 shows the TSI values for the three processing groups. Clearly, the TSI value for MP was the highest and tended to rise with the extension of time, showing that the dynamic instability of the emulsion increased and apparent stratification appeared. The TSI values for MP-CPI and MP-CPI_DBD_ gradually decreased, and more stable emulsions were produced by mixing proteins, which might be related to the conformation of the protein and the adsorption capacity of the protein at the oil–water interface (explained in the subsequent results).

### 3.3. Optical Microscopy and Particle Size

**Figure 2:**
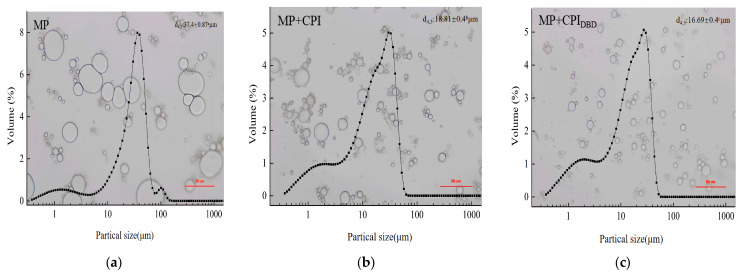
Optical microscopy images and particle size distributions of emulsions prepared from myofibrillar protein (MP) and from untreated and dielectric barrier discharge (DBD)-treated CPI-MP complexes. (**a**) MP: myofibrillar protein; (**b**) MP + CPI: addition of native CPI; (**c**) MP + CPI_DBD_: addition of DBD-treated CPI. ^a–c^ Different superscript letters above the data (d_4,3_) represent that the means are significant differences (*p* < 0.05).

Compared with MP, the MP-CPI_DBD_ emulsion showed smaller droplets; the particle size distribution also confirmed a reduction in the emulsion particle size that was consistent with the change in the droplet size. The emulsion produced from MP presented larger-scale droplets, and the size of droplets was not uniform. This shows that coalescence and flocculation phenomena might have occurred, resulting in the formation of emulsion stratification. Figure 2b shows the emulsion formed from MP-CPI. It can be seen that there were still relatively large droplets, and some small droplets and aggregation appeared in the composite system. This phenomenon might be due to the dense structure of the natural CPI, which did not interact well with MP, and the oil droplets were not adequately coated. The droplet size shown in Figure 2c was more uniform, as was the dispersion. In our previous research, we found that the solubility, emulsification property, and anti-oil drop flocculation property of CPI were improved after plasma treatment [18,33]. This might be an important factor affecting the stability of the emulsion [34]. Figure 2 shows the changes in particle size of the emulsions from the three treatment groups. It can be clearly seen that the emulsion prepared from MP had the largest particle size, at 32.40 μm. However, the particle size of the emulsion prepared from the mixed proteins was smaller (*p* < 0.05), and the particle size of MP-CPI_DBD_ was the smallest, at 16.69 μm. This might be due to the interaction of the two proteins to form a complex, which produces steric hindrance and inhibits the aggregation of oil droplets. On the other hand, it might be related to the decrease in the particle size of CPI after plasma modification. Wang et al. [35] found that the addition of 0.05% polysaccharides derived from soy hulls to soybean protein isolate resulted in the formation of an emulsion with smaller particle size. Furthermore, the emulsion exhibited a narrower and more concentrated distribution of droplets compared to the emulsion without soy hull polysaccharides.

### 3.4. CI

**Figure 3:**
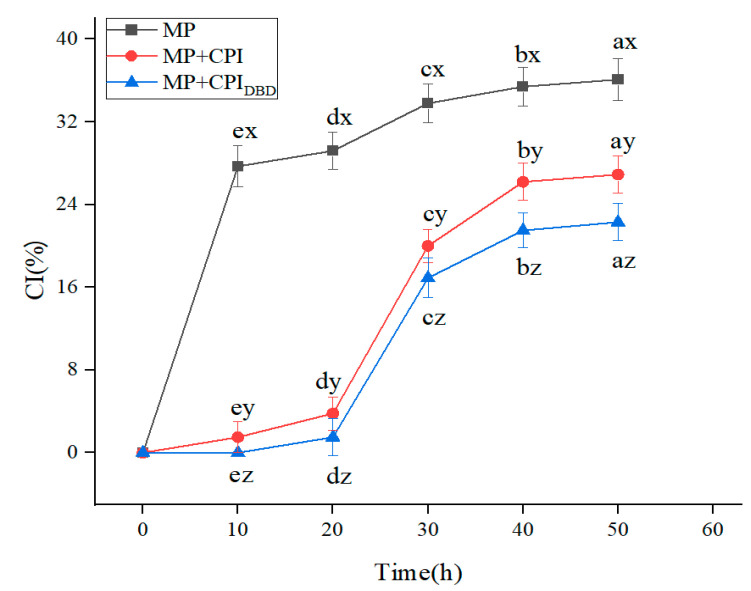
Creaming index values of emulsions prepared from myofibrillar protein (MP) and from untreated and dielectric barrier discharge (DBD)-treated CPI-MP complexes. MP: myofibrillar protein; MP + CPI: addition of native CPI; MP + CPI_DBD_: addition of DBD-treated CPI. ^a–e^ Different superscript letters in the same treatment represent significant differences (*p* < 0.05). ^x–z^ Different superscript letters indicate significant differences among the means within the same storage time.

**Figure 4:**
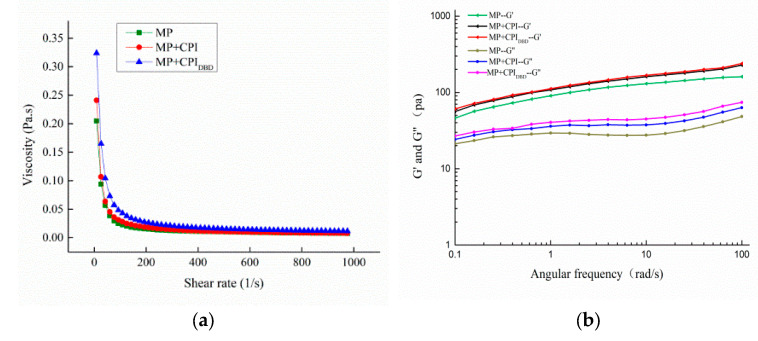

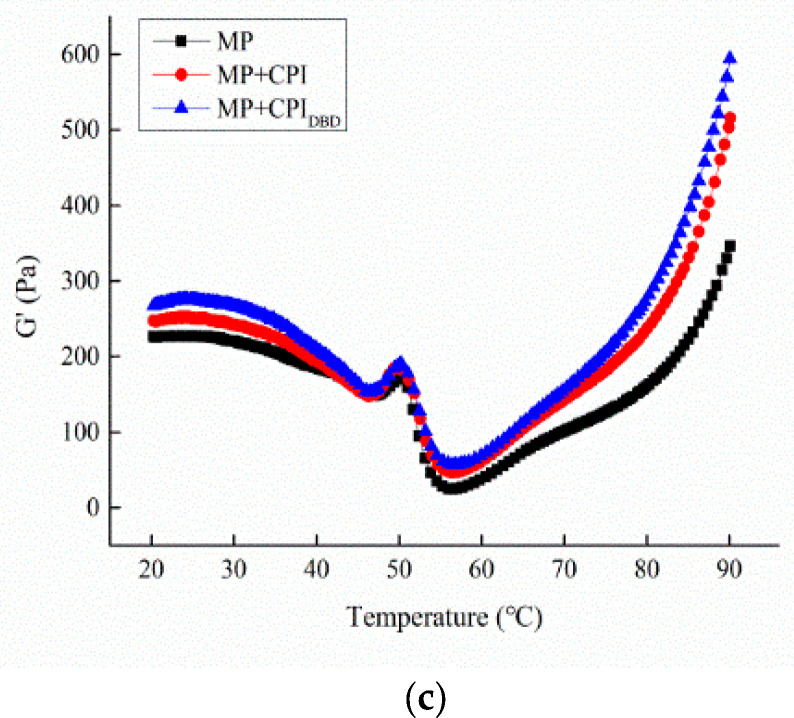
Rheological properties of emulsions prepared from myofibrillar protein (MP) and from untreated and dielectric barrier discharge (DBD)-treated CPI-MP complexes. (**a**) Viscosity; (**b**) frequency sweep of emulsions; (**c**) temperature sweep of emulsions. MP: myofibrillar protein; MP + CPI: addition of native CPI; MP + CPI_DBD_: addition of DBD-treated CPI.

Flocculation and coalescence between emulsion droplets accelerate the delamination of the emulsion, and a transparent layer appears at the bottom of the sample [6,8]. In this experiment, mild conditions were used for the preparation of the emulsions (they were prepared without high-pressure homogenization), which had an impact on the stability of the emulsions, and they showed apparent stratification in the process of short-term storage (Figure 3). After 12 h of storage, the emulsion prepared from MP was stratified, while the emulsion prepared from MP-CPI showed slight discontinuity, and the emulsion prepared from MP-CPI_DBD_ was relatively stable; the CI of all the emulsions tended to be stable after 24 h of storage. It may be that the addition of CPI increased the viscosity of the continuous phase (Figure 4a), the droplet migration was limited, and the emulsion stability was eventually improved. Yakoub et al. [36] found that a lower decrease in the value of the emulsion precipitation index indicated that a strong viscoelastic film had formed at the interface and that the emulsion was more stable.

### 3.5. Rheological Property Analysis

#### 3.5.1. Apparent Viscosity

The viscosity of an emulsion is an important parameter of the emulsion’s stability. The dynamic rheological properties of the emulsions were measured using a rotational rheometer to characterize the viscoelasticity of the samples. From Figure 4a, we can see that the apparent viscosity of all emulsions decreased rapidly at the beginning and then gradually became flat and stable with increasing shear rate in the range of 0.1–1000 s^−1^, which indicated that shear dilution occurred and displayed non-Newtonian pseudo-plastic behaviors. At the same time, the viscosity of the emulsion with added CPI increased, which might be due to the interaction between the proteins. Plasma treatment altered the structure of the CPI, increased the flexibility of the protein molecules, and facilitated the interaction with MP. In addition, when the apparent viscosity of a system is high, the movement rate of oil droplets is slowed, which reduces the collision frequency and reduces the degree of coalescence of oil droplets [37,38]. Ma et al. [39] found that the addition of xanthan gum to a whey protein citral system increased the viscosity of the continuous phase of the emulsion and enhanced the spatial hindrance of aggregation between oil droplets, having a significant stabilizing effect on the emulsion.

#### 3.5.2. Frequency Sweep Testing

The results in Figure 4b show that within the frequency test range, both the storage modulus (G′) and the loss modulus (G″) of the emulsion gradually increased in the angular frequency scanning mode. The G′ was higher than the G″, and there was no crossover between them, illustrating that the elastic gel property manifested and the emulsion formed a weak gel structure [40]. It can also be seen that the emulsion prepared from MP-CPI_DBD_ had higher elasticity than the others. This was attributed to the plasma treatment that enhanced the surface hydrophobicity of the CPI and enhanced the interactions between the proteins in the hybrid system, which allowed for the creation of highly viscous and elastic interfaces to achieve optimal stability of the interfacial layer [41]. Zhao et al. [42] reported higher G’ than G” at various protein concentrations, indicating a more elastic protein structure.

#### 3.5.3. Temperature Sweep Measurement

The change in the storage modulus (G′) as the temperature increased from 20 °C to 90 °C is shown in Figure 4c. For MP, the value of G′ started to decrease gradually from 20 °C to 47 °C, then it increased quickly and peaked at 50 °C. Subsequently, the value of G’ decreased sharply to its minimum value at 56 °C and steadily increased with further heating to 90 °C. The G′ values of all emulsions decreased slightly from 20 °C to 47 °C. A similar result was observed by Li et al. [21], who found that this could be attributed to the initial weak interactions formed by the protein aggregates prior to heating. With the temperature increase, the myosin head swelled and the protein molecules interacted with each other to form an elastic network, leading to an increase in the G’ value. When the temperature reached 54 °C, the myosin was denatured, which contributed to the destruction of the network and the downward trend of G’. Subsequently, the denaturation and ligation of most of the proteins induced a gel network that led to a significant increase in G’ until the end [43]. The mixed system MP-CPI_DBD_ had the highest curve from beginning to end, indicating a stronger gel structure, which was due to the active substance in the plasma expanding the molecular structure of the CPI, exposing more hydrophobic groups to enhance their hydrophobic interaction, resulting in higher elasticity and higher G’ values. Zhou et al. [44] also discovered that a smaller particle size and uniform dispersion of oil droplets are more conducive to filling the gel network structure, resulting in an increase in the G’ value.

### 3.6. Interfacial Tension

**Figure 5:**
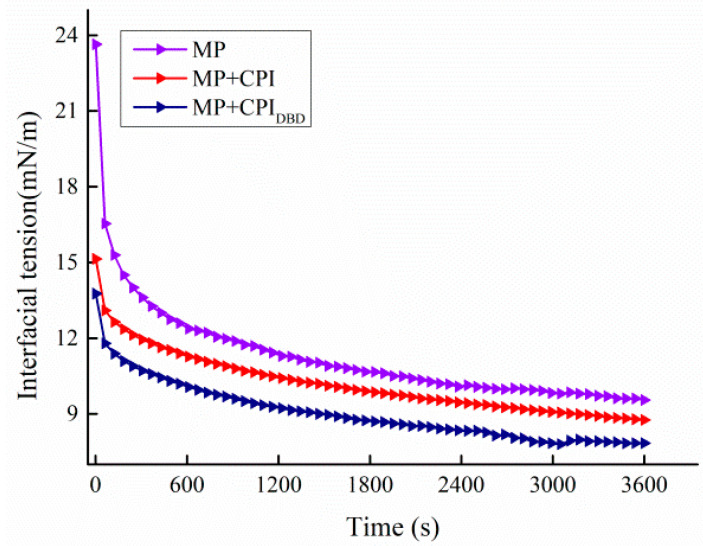
Interfacial tension of emulsions prepared from myofibrillar protein (MP) and from untreated and dielectric barrier discharge (DBD)-treated CPI-MP complexes. A: Viscosity; B: frequency sweep of emulsions; C: temperature sweep of emulsions. MP: myofibrillar protein; MP + CPI: addition of native CPI; MP + CPI_DBD_: addition of DBD-treated CPI.

Figure 5 shows the changes in interfacial tension in the emulsions from the three different groups. In general, the change trends of all curves were as follows: The interfacial tension of the emulsion decreased from 23.64 to 15.14 and 13.76 mN/m at the start, and the interfacial tension was 9.55, 8.76, and 7.84 mN/m at the final stage, respectively. First, the interfacial tension gradually decreased to a steady state with the extension of time due to the co-existence of polar and non-polar groups of amino acid residues in the protein molecules, which connected the water and oil through their hydrophilic and hydrophobic properties, reducing the surface tension of the two phases [45,46]. Second, the interfacial tension went through two stages: rapid decrease and slow decrease. The interfacial tension still decreased slowly during the 60 min adsorption time, which indicated that the protein could not reach equilibrium at the oil–water interface in this time period [47]. This is due to the fact that proteins are surface-active substances with large molecular weights, and the adsorption process at the interface was relatively slow; therefore, they might take longer to reach the final equilibrium state [48]. Finally, the interfacial tension in the mixed protein systems was lower and maintained the same trend during the whole process. This indicated that the mixed protein was more conducive to diffusion to the surface of the oil droplets and penetration at the O/W interface, which resulted in more effective adsorption at the oil–water interface, thus reducing the interfacial tension. Rafe et al. [49] found that the foam stability of BLG/κ-carrageenan mixtures increased with increasing κ-carrageenan concentration and decreased surface tension at increasing pH 7 stability, indicating a synergistic effect on interfacial adsorption and foam stabilization. Sun et al. [44] found that when xanthan gum and whey protein isolate were adsorbed together on the surface of oil droplets, the thickness of the interfacial film at the oil–water interface was increased and the interfacial tension was effectively reduced, suggesting that the elevated viscosity of the aqueous phase could also be connected to the interfacial tension in some way. The results further confirmed that the emulsions were more stable when the interfacial tension was low [50,51].

### 3.7. Interfacial Protein Content

**Figure 6:**
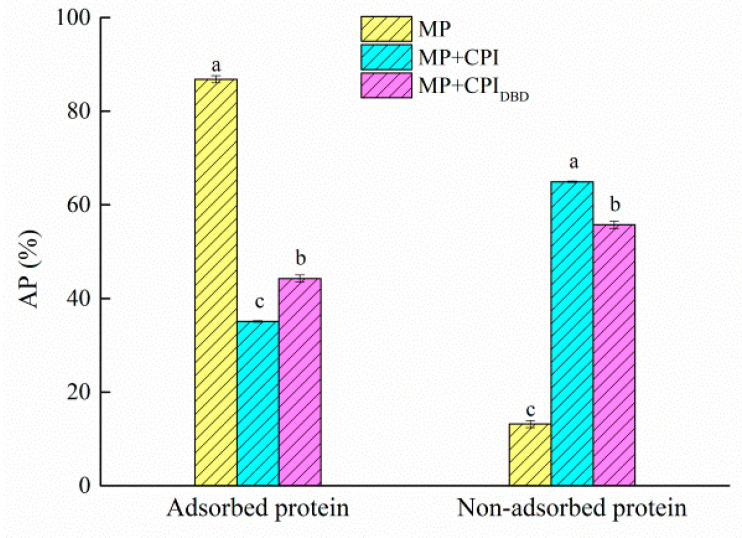
Adsorbed and non-adsorbed fractions of the myofibrillar protein (MP) and the untreated and dielectric barrier discharge (DBD)-treated CPI-MP complex emulsions. Adsorbed protein is located in the emulsion layer; non-adsorbed protein is located in the aqueous layer. ^a–c^ Different letters in the same protein groups represent significant differences (*p* < 0.05).

Protein adsorption is a complex dynamic process that is affected by the temperature, protein concentration, protein molecular size, and protein surface hydrophobicity [52]. As shown in Figure 6, the content of interfacial protein in the single system was as high as 86.82%, while those in the mixed systems were 35.05% and 44.27%. The reasons for the decrease in interfacial protein content in the different systems might be as follows: (1) In this study, the concentration of MP was selected as 6 mg/mL. As a result, most of the proteins could be adsorbed on the interface, there was competitive adsorption between the two proteins, and the binding space and opportunity between the interface and the new protein were reduced [22]. (2) The chickpea protein isolate had an isoelectric point of about 5, whereas the pH of the aqueous phase was about 7. This enhanced the electrostatic repulsion between the protein molecules adsorbed on the interface and prevented the protein molecules from adsorbing firmly on the surface of the oil droplets [53]. (3) It is possible that the protein aggregation in the emulsion decreased the content of interfacial protein through a steric hindrance effect. For the mixed systems, the interfacial content was only about 35–45%, indicating that there were still many non-adsorbed protein molecules in the aqueous solution. However, the interface content of MP-CPI_DBD_ was higher than that of MP-CPI, which was consistent with our previous results. Plasma treatment of the chickpea protein isolates changed the structure and improved the flexibility of the protein, which was beneficial for intermolecular interactions at the interface. It has been proved that a higher flexibility of protein results in more hydrophobic amino acids adsorbed at the water–oil interface, forming a stable interface film [54].

### 3.8. Interfacial Protein Composition

**Figure 7:**
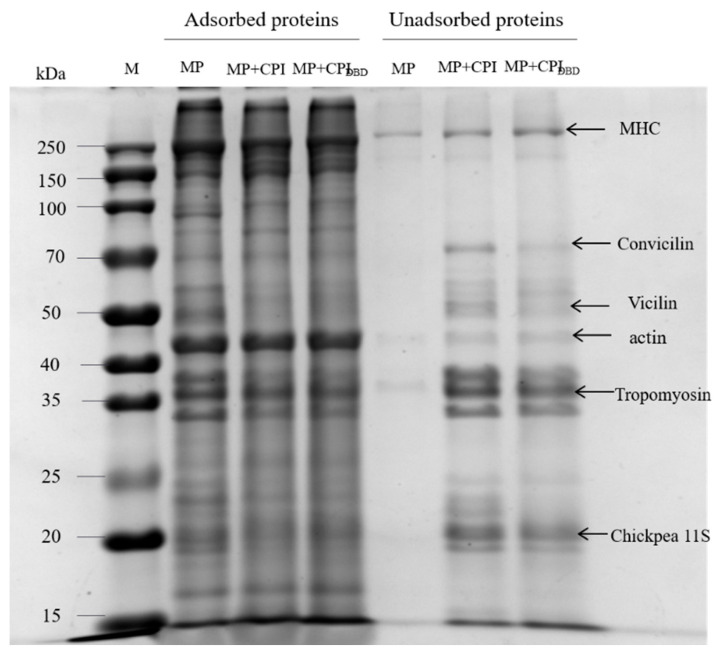
Adsorbed proteins and non-adsorbed proteins in emulsions prepared from myofibrillar protein (MP) and from untreated and dielectric barrier discharge (DBD)-treated CPI-MP complexes. Sodium dodecyl sulfate-polyacrylamide gel electrophoresis of adsorbed proteins and non-adsorbed proteins in emulsions. Lane 1: M indicates the standard marker (kDa). MP: myofibrillar protein; MP + CPI: addition of native CPI; MP + CPI_DBD_: addition of DBD-treated CPI.

Figure 7 shows the electrophoretic patterns of the interface proteins under reduction conditions (by adding DL-Dithiothreitol, DTT). We can see that the bands of MHC and actin became thicker in the bands of the non-adsorbed proteins, while the bands of various subunits of chickpea isolate became clearer in the bands of the adsorbed proteins, which was in accordance with the above calculations of adsorbed protein concentrations. Deng et al. [55] showed that MP is made up of myosin heavy chain bands (MHC, 220 kDa), actin (42 kDa), and some low-intensity bands, including tropomyosin (33–37 kDa), troponin C (19 kDa), and myosin light chain (MLC, 16 kDa). CPI showed a variety of polypeptide subunits between 25 and 75 kDa, specifically including a subunit of 70 kDa, corresponding to convicilin, and a subunit around 50 kDa, possibly defined as Vicilin, including three polypeptide subunits (α-, β-, and γ-subunits) with a molecular weight between 43 and 53 kDa. The peptide subunits around 37 and 25 kDa could be attributed to the acidic and basic subunits of legumes, respectively [36]. From this, we hypothesized that the addition of chickpea isolate reduced the effective adsorption component of MP, while the subunit of chickpea isolate replaced part of the subunit of MP and preferentially adsorbed on the interface, which was related to the size of the protein molecules. To some extent, the two proteins also interacted with each other, but rather than adsorbing synergistically, they were more likely to be adsorbed competitively at the interface [56]. Mourtzinos et al. [57] studied the competitive emulsification between soy protein isolate and MP, and the results showed that soy protein isolate competitively replaced MP on the fat surface.

### 3.9. Microstructure

**Figure 8:**
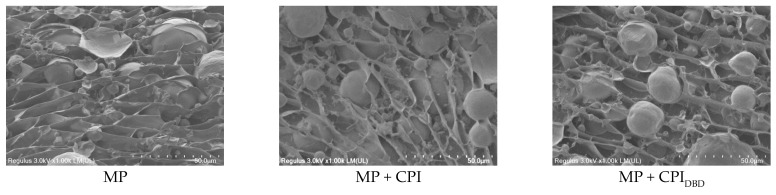
Scanning electron microscopy images of emulsions prepared from myofibrillar protein (MP) and from untreated and dielectric barrier discharge (DBD)-treated CPI-MP complexes. MP: myofibrillar protein; MP + CPI: addition of native CPI; MP + CPI_DBD_: addition of DBD-treated CPI.

We can see from Figure 8 that the emulsion droplets were spherical and had a relatively smooth surface. Compared with those of the control group, the droplets of the composite emulsion were smaller in size and more evenly dispersed [58]. The microstructure also tended to be more compact. Our study proved that the chickpea protein structure was expanded after plasma treatment for 4 min, which made it easier for MP molecules to interact with each other and be adsorbed at the oil–water interface to prevent the aggregation of oil droplets [18]. Consistent with the results of Ghorbani-HasanSaraei et al. [59], it was observed via SEM that the droplet dispersion of the treated samples was more uniform. The results were consistent with the particle size and emulsifying activity.

## 4. Conclusions

The stability of mixed protein systems in emulsions was studied herein. The results showed that the addition of chickpea protein isolate reduced the particle size of the emulsion, which led to smaller and more uniformly distributed emulsion droplets and improved the emulsion’s stability. Compared with MP and MP-CPI emulsions, the MP + CPI_DBD_ emulsion presented increased emulsion viscosity, reduced droplet size, and greater stability due to the prevention of droplet aggregation. As an emulsifier, it not only showed the highest G’ value, but also helped to form better O/W emulsion gel elasticity. In conclusion, DBD-treated CPI increased the application of the mixed protein system to increase the stability of the resulting emulsion, which is expected to provide a reference for the research and application of emulsion products. On this basis, the application of some emerging technologies, such as ultrasound, plasma, and high pressure, is also an important research direction to improve the function and properties of the mixed protein system.

## Figures and Tables

**Table 1:** Emulsifying activity index and emulsion stability index values of untreated and dielectric barrier discharge (DBD)-treated CPI-MP complexes.

Sample	Emulsifying Activity Index (m^2^/g)	Emulsion Stability Index (%)
MP	55.17 ± 0.18 ^c^	66.31 ± 1.09 ^c^
MP + CPI	70.96 ± 1.12 ^b^	94.74 ± 0.49 ^b^
MP + CPI_DBD_	74.99 ± 1.45 ^a^	99.87 ± 0.37 ^a^

MP: myofibrillar protein; MP + CPI: addition of native CPI; MP + CPI_DBD_: addition of DBD-treated CPI. ^a–c^ Different superscript letters in the same column represent significant differences (*p* < 0.05).

## Data Availability

Data are contained within the article.

## References

[1] Chen X., Tume R.K., Xu X.L., Zhou G.H. (2017). Solubilization of myofibrillar proteins in water or low ionic strength media: Classical techniques, basic principles, and novel functionalities. Crit. Rev. Food Sci. Nutr..

[2] Sun X.D., Holley R.A. (2011). Factors influencing gel formation by myofibrillar proteins in muscle foods. Compr. Rev. Food Sci. Food Saf..

[3] Chang H.J., Xu X.L., Zhou G.H., Li C.B., Huang M. (2012). Effects of characteristics changes of collagen on meat physicochemical properties of beef semitendinosus muscle during ultrasonic processing. Food Bioprocess Technol..

[4] Feng Y., Xu J., Yu D., Kong B., Liu Q. (2019). Recent advances in the application of emulsion gels as fat replacers in meat products. Shipin Kexue/Food Sci..

[5] Barbut S. (1995). Importance of fat emulsification and protein matrix characteristics in meat batter stability. J. Muscle Foods.

[6] Wang J., Han X., Li T., Yu G. (2020). Mechanism and application of emulsifiers for stabilizing emulsions: A review. Shipin Kexue/Food Sci..

[7] Alvarez D., Xiong Y.L., Castillo M., Payne F.A., Garrido M.D. (2012). Textural and viscoelastic properties of pork frankfurters containing canola-olive oils, rice bran, and walnut. Meat Sci..

[8] Bouyer E., Mekhloufi G., Rosilio V., Grossiord J., Agnely F. (2012). Proteins, polysaccharides, and their complexes used as stabilizers for emulsions: Alternatives to synthetic surfactants in the pharmaceutical field. Int. J. Pharm..

[9] Xu D.X., Aihemaiti Z., Cao Y.P., Teng C., Li X.T. (2016). Physicochemical stability, microrheological properties and microstructure of lutein emulsions stabilized by multilayer membranes consisting of whey protein isolate, flaxseed gum and chitosan. Food Chem..

[10] Jiang J., Zhu B., Liu Y.F., Xiong Y.L. (2014). Interfacial structural role of pH-shifting processed pea protein in the oxidative stability of oil/water emulsions. J. Agric. Food Chem..

[11] Ji J., Zhang J.P., Chen J.S., Wang Y.L., Dong N., Hu C.L., Chen H.P., Li G., Pan X., Wu C.B. (2015). Preparation and stabilization of emulsions stabilized by mixed sodium caseinate and soy protein isolate. Food Hydrocoll..

[12] Jukanti A.K., Gaur P.M., Gowda C.L.L., Chibbar R.N. (2012). Nutritional quality and health benefits of chickpea (*Cicer arietinum* L.): A review. Br. J. Nutr..

[13] Wang S., Chelikani V., Serventi L. (2018). Evaluation of chickpea as alternative to soy in plant-based beverages, fresh and fermented. LWT.

[14] Zhang Y., Huang X., Zeng X., Li L., Jiang Y. (2023). Preparation, functional properties, and nutritional evaluation of chickpea protein concentrate. Cereal Chem..

[15] Nunes L., Tavares G.M. (2019). Thermal treatments and emerging technologies: Impacts on the structure and techno-functional properties of milk proteins. Trends Food Sci. Technol..

[16] Bußler S., Steins V., Ehlbeck J., Schlueter O. (2015). Impact of thermal treatment versus cold atmospheric plasma processing on the techno-functional protein properties from *Pisum sativum* ‘Salamanca’. J. Food Eng..

[17] Ji H., Dong S., Han F., Li Y.T., Chen G.Y., Li L., Chen Y. (2018). Effects of dielectric barrier discharge (DBD) cold plasma treatment on physicochemical and functional properties of peanut protein. Food Bioprocess Technol..

[18] Li K., Tian J.F., Zheng S.Y., He Y.Y., Xiang Q.S., Bai Y.H. (2021). Effects of plasma on solubility and emulsifying properties of chickpea protein isolates. Trans. Chin. Soc. Agric. Eng..

[19] Mession J.L., Roustel S., Saurel R. (2015). Interactions in casein micelle-Pea protein system (part I): Heat-induced denaturation and aggregation. Food Hydrocoll..

[20] Wang Y.T., Wang Y.J., Li K., Bai Y.H., Li B., Xu W. (2020). Effect of high intensity ultrasound on physicochemical, interfacial and gel properties of chickpea protein isolate. LWT.

[21] Li K., Fu L., Zhao Y.Y., Xue S.W., Wang P., Xu X.L., Bai Y.H. (2020). Use of high-intensity ultrasound to improve emulsifying properties of chicken myofibrillar protein and enhance the rheological properties and stability of the emulsion. Food Hydrocoll..

[22] Li L., Xiong Y.L. (2021). Competitive adsorption and dilatational rheology of pork myofibrillar and sarcoplasmic proteins at the O/W emulsion interface. Food Hydrocoll..

[23] Li K., Li Y., Liu C.L., Fu L., Zhao Y.Y., Zhang Y.Y., Wang Y.T., Bai Y.H. (2020). Improving interfacial properties, structure and oxidative stability by ultrasound application to sodium caseinate prepared pre-emulsified soybean oil. LWT.

[24] Jahromi M., Niakousari M., Golmakani M.T., Ajalloueian F., Khalesi M. (2020). Effect of dielectric barrier discharge atmospheric cold plasma treatment on structural, thermal and techno-functional characteristics of sodium caseinate. Innov. Food Sci. Emerg. Technol..

[25] Ma C.C., Jiang W., Chen G.P., Wang Q.K., McClements D.J., Liu X.B., Liu F.G., Ngai T. (2020). Sonochemical effects on formation and emulsifying properties of zein-gum arabic complexes. Food Hydrocoll..

[26] O’Sullivan J., Murray B., Flynn C., Norton I. (2016). The effect of ultrasound treatment on the structural, physical and emulsifying properties of animal and vegetable proteins. Food Hydrocoll..

[27] Mathé C., Devineau S., Aude J.C., Lagniel G., Chedin S., Legros V., Mathon M.H., Renault J.P., Pin S., Boulard Y. (2013). Structuraldeterminants for protein adsorption/non-adsorption to silica surface. PLoS ONE.

[28] Zeng G.Z., Zhao Z.H., Zhou Z.P., Deng Y.Y., Wei Z.C., Zhang Y., Tang X.J., Liu G., Li P., Zhang M.W. (2023). Effects of red removal and ultrasonic treatment on peanut oil extraction and emulsification characteristics. China Oils Fats.

[29] Wu C., Wang T., Ren C., Ma W., Wu D., Xu X., Wang L., Du M. (2021). Advancement of food-derived mixed protein systems: Interactions, aggregations, and functional properties. Compr. Rev. Food Sci. Food Saf..

[30] Feng J., Xiong Y.L. (2003). Interaction and functionality of mixed myofibrillar and enzyme-hydrolyzed soy proteins. J. Food Sci..

[31] Zhang X.Y., Zhang S., Xie F.Y., Han L., Li L., Jiang L.Z., Qi B.K., Li Y. (2020). Soy/whey protein isolates: Interfacial properties and effects on the stability of oil-in-water emulsions. J. Sci. Food Agric..

[32] Wang Q.L., Jin Y., Xiong Y.L. (2018). Heating-aided pH shifting modifies hemp seed protein structure, cross-linking, and emulsifying properties. J. Agric. Food Chem..

[33] Li K., Jia S.X., Tian J.F., Wu L.L. (2023). Emulsifying characteristics of chickpea powder. Food Res. Dev..

[34] Wen X., Li M., Ersan S., Hu R., Geng N., Ni Y.Y. (2018). Formation and stability of zeaxanthin dipalmitate emulsions. Trans. Chin. Soc. Agric. Mach..

[35] Wang S.N., Yang J.J., Shao G.Q., Qu D.N., Zhao H.K., Yang L.N., Zhu L.J., He Y.T., Liu H., Zhu D.S. (2020). Soy protein isolated-soy hull polysaccharides stabilized O/W emulsion: Effect of polysaccharides concentration on the storage stability and interfacial rheological properties. Food Hydrocoll..

[36] Ladjal-Ettoumi Y., Boudries H., Chibane M., Romero A. (2016). Pea, chickpea and lentil protein isolates: Physicochemical characterization and emulsifying properties. Food Biophys..

[37] Taha A., Hu T., Zhang Z., Bakry A.M., Khalifa I., Pan S., Hu H. (2018). Effect of different oils and ultrasound emulsification conditions on the physicochemical properties of emulsions stabilized by soy protein isolate. Ultrason. Sonochemistry.

[38] Diao X.Q., Guan H.N., Zhao X.X., Chen Q., Kong B.H. (2016). Properties and oxidative stability of emulsions prepared with myofibrillar protein and lard diacylglycerols. Meat Sci..

[39] Bengoechea C., Romero A., Aguilar J.M., Cordobes F., Guerrero A. (2010). Temperature and pH as factors influencing droplet size distribution and linear viscoelasticity of O/W emulsions stabilised by soy and gluten proteins. Food Hydrocoll..

[40] Ma L., Mao D.B. (2019). Preparation of citral emulsion stabilized by chickpea whey protein. Food Sci. Technol..

[41] Feng T.T., Wang X.W., Wang X.J., Xia S.Q., Huang Q.R. (2021). Plant protein-based antioxidant pickering emulsions and high internal phase pickering emulsions against broad pH range and high ionic strength: Effects of interfacial rheology and microstructure. LWT.

[42] Zhao X., Wu T., Xing T., Xu X.L., Zhou G.H. (2019). Rheological and physical properties of O/W protein emulsions stabilized by isoelectric solubilization/precipitation isolated protein: The underlying effects of varying protein concentrations. Food Hydrocoll..

[43] Gao T.X., Zhao X., Li R., Bassey A., Bai Y., Ye K., Deng S.L., Zhou G.H. (2022). Synergistic effects of polysaccharide addition-ultrasound treatment on the emulsified properties of low-salt myofibrillar protein. Food Hydrocoll..

[44] Zhou X., Chen H., Lyu F., Lin H., Zhang Q., Ding Y. (2019). Physicochemical properties and microstructure of fish myofibrillar protein-lipid composite gels: Effects of fat type and concentration. Food Hydrocoll..

[45] Li K., Li S.Y., He Y.Y., Wang Y.Q., Zhang Y.X., Zhao Y.Y., Du M.T., Wang Y., Wang Y.T., Bai Y.H. (2022). Application of ultrasound-assisted alkaline extraction for improving the solubility and emulsifying properties of pale, soft, and exudative (PSE)-like chicken breast meat protein isolate. LWT.

[46] Shao P., Feng J.R., Sun P.L., Ritzoulis C. (2019). Improved emulsion stability and resveratrol encapsulation by whey protein/gum arabic interaction at oil-water interface. Int. J. Biol. Macromol..

[47] Lam R.S., Nickerson M.T. (2013). Food proteins: A review on their emulsifying properties using a structure–function approach. Food Chem..

[48] Sun C.H., Gunasekaran S. (2010). Rheology and oxidative stability of whey protein isolate-stabilized menhaden oil-in-water emulsions as a function of heat treatment. J. Food Sci..

[49] Rafe A., Glikman D., Rey N.G., Haller N., Kulozik U., Braunschweig B. (2022). Structure-property relations of β-lactoglobulin/κ-carrageenan mixtures in aqueous foam. Colloids Surf. A Physicochem. Eng. Asp..

[50] Derkatch S.R., Levachov S.M., Kuhkushkina A.N., Novosyolova N.V., Kharlov A.E., Matveenko V.N. (2007). Rheological properties of concentrated emulsions stabilized by globular protein in the presence of nonionic surfactant. Colloids Surf. A Physicochem. Eng. Asp..

[51] Sui X., Bi S., Qi B., Wang Z., Zhang M., Li Y. (2017). Impact of ultrasonic treatment on an emulsion system stabilized with soybean protein isolate and lecithin: Its emulsifying property and emulsion stability. Food Hydrocoll..

[52] Kim Y.H., Nikolov A.D., Wasan D.T., Diaz-Arauzo H., Shelly C.S. (1996). Demulsification of water in crude oil emulsions: Effects of film tension, elasticity, diffusivity and interfacial activity of demulsifier individual components and their blends. J. Dispers. Sci. Technol..

[53] Murray B.S., Dickinson E. (1996). Interfacial rheology and the dynamic properties of adsorbed films of food proteins and surfactants. Food Sci. Technol. Int. Tokyo.

[54] Valerio M., Colosimo A., Conti F., Giuliani A., Grottesi A., Manetti C., Zbilut J.P. (2005). Early events in protein aggregation: Molecular flexibility and hydrophobicity/charge interaction in amyloid peptides as studied by molecular dynamics simulations. Proteins Struct. Funct. Bioinform..

[55] Deng X.R., Ma Y.G., Lei Y.D., Zhu X.R., Zhang L.F., Hu L., Lu S.L., Guo X., Zhang J. (2021). Ultrasonic structural modification of myofibrillar proteins from *Coregonus peled* improves emulsification properties. Ultrason. Sonochem..

[56] Acton J.C., Saffle R.L. (1972). Emulsifying capacity of muscle protein: Phase volumes at emulsion collapse. J. Food Sci..

[57] Mourtzinos I., Kiosseoglou V. (2005). Protein interactions in comminuted meat gels containing emulsified corn oil. Food Chem..

[58] Wang Y., Wang S., Li R., Wang Y., Xiang Q., Qiu S., Xu W., Bai Y. (2022). Synergistic effect of corn fiber gum and chitosan in stabilization of oil in water emulsion. LWT.

[59] Ghorbani-HasanSaraei A., Rafe A., Shahidi S.A., Atashzar A. (2019). Microstructure and chemorheological behavior of whipped cream as affected by rice bran protein addition. Food Sci. Nutr..

